# The niche‐centric view: Redefining inflammatory disease beyond molecular signatures

**DOI:** 10.1111/jdv.70301

**Published:** 2026-02-25

**Authors:** Jennifer Lavina Ngo, Curdin Conrad

**Affiliations:** ^1^ Department of Dermatology Rizal Medical Center Pasig City Philippines; ^2^ Department of Dermatology Lausanne University Hospital (CHUV) and University of Lausanne Lausanne Switzerland

The identification of key pathogenic cytokines and association of certain immune pathways with distinct inflammatory skin diseases have paved the way for targeted therapies and novel diagnostic tools.^1^ While bulk transcriptomic analyses represent a cost‐effective and accessible method to measure average gene expression within a lesion, this approach obscures key layers of information: cell type heterogeneity and potentially crucial spatial organization. Inflammation is not evenly distributed; rather, it is tightly compartmentalized within skin lesions. These microenvironmental niches can influence which cells interact, which structures are involved, and how accessible they are to therapeutic intervention.

Jiang and colleagues contend that lesions should be regarded as collections of inflammatory compartments rather than as uniformly inflamed tissue.^2^ A niche constitutes more than a mere collection of cells. It reflects a spatially organized circuit, composed of immune cells, vessels, stroma and epithelium, that contributes to disease memory and recurrence and enables chronic disease progression. Using hidradenitis suppurativa and psoriasis as an example, the authors elucidate how spatial transcriptomics and the concept of inflammatory niches help to understand why these two diseases—despite sharing common key inflammatory pathways and pathogenic cytokines—display different response rates to targeted therapies.^2^

The authors present a persuasive argument that these spatial patterns function as determinants of skin disease. Indeed, this concept is already reshaping our understanding of other inflammatory conditions. Recent spatial data in primary cicatricial alopecia reveal a hostile, follicle‐centric environment characterized by immune cells actively compromising epithelial immune privilege.^3^ These distinctions are not solely superficial; they represent discrete microanatomical circuits. The authors also emphasize the presence of compartmentalized cytotoxic circuits at the dermo‐epidermal junction in vitiligo, along with growing neuro‐immune influences that modulate dendritic cell function and local CD8^+^ T cell activity.^2^ With the identification of Cathepsin S‐expressing melanocytes situated within specific compartments alongside antigen‐presenting cells,^4^ this finding transforms the inflammatory niche from a promising discovery into a viable therapeutic target: to identify and interrupt circuits within defined tissue regions.

The concept of the inflammatory niche raises the bar for personalized medicine, and spatial transcriptomics provides unprecedented insight into tissue‐level heterogeneity in inflammatory skin diseases. By integrating gene expression with tissue localization, spatial transcriptomics enables direct correlation between molecular profile and histopathologic structure, allowing us to accurately map the structural organization of tissue inflammation. However, a note of caution is still warranted. Spatial omics has limitations: low‐abundance transcripts may be overlooked, cell identities often rely on marker gene inference, and cost and technical demands remain barriers to broader implementation.^5^ It will be interesting to see if artificial intelligence‐powered analyses can soon reverse the current paradigm and infer specific immune pathways and inflammatory niches from regular histopathology alone, thereby bringing precision medicine closer to everyday clinical routine. Ultimately, whether treatment selection based on niche‐specific targets can improve outcomes—and whether the concept of inflammatory niches will evolve into a practical tool for therapeutic decision‐making—remains an open and important question.

## FUNDING INFORMATION

The author(s) received no financial support for the research, authorship and/or publication of this article.

## CONFLICT OF INTEREST STATEMENT

JLN: Speaker honoraria—Novartis and Sun Pharmaceuticals. CC: Scientific adviser and/or clinical study investigator—AbbVie, Actelion, Almirall, Amgen, Boehringer Ingelheim, Bristol Myers Squibb, Celgene, Eli Lilly, Galderma, Incyte, Jiangsu Hengrui, Johnson & Johnson, LEO Pharma, MSD, Novartis, Pfizer, Samsung, Sanofi, Takeda and UCB.

## ETHICS STATEMENT

Not applicable.

## Data Availability

Data sharing is not applicable to this article as no new data were created or analyzed in this study.
